# Correction: Restorative biodegradable two‑layered hybrid microneedles for melanoma for melanoma photothermal/chemo co-therapy and wound healing

**DOI:** 10.1186/s12951-026-05126-2

**Published:** 2026-10-06

**Authors:** Yue Shan, Bowen Tan, Min Zhang, Xi Xie, Jinfeng Liao

**Affiliations:** State Key Laboratory of Oral Diseases, National Clinical Research Centre for Oral Diseases, West China Hospital of Stomatology, Sichuan University, Chengdu, 610041 People’s Republic of China

**Correction: Journal of Nanobiotechnology (2022) 20:238**

**https://doi.org/10.1186/s12951-022-01426-5**

In this article, an incorrect version of Fig. 7(A) and Fig. 8(A) were included. The old incorrect and correct versions of Figs. 7(A) and 8(A) are presented below.

Incorrect Fig. 7.

**Figure:**
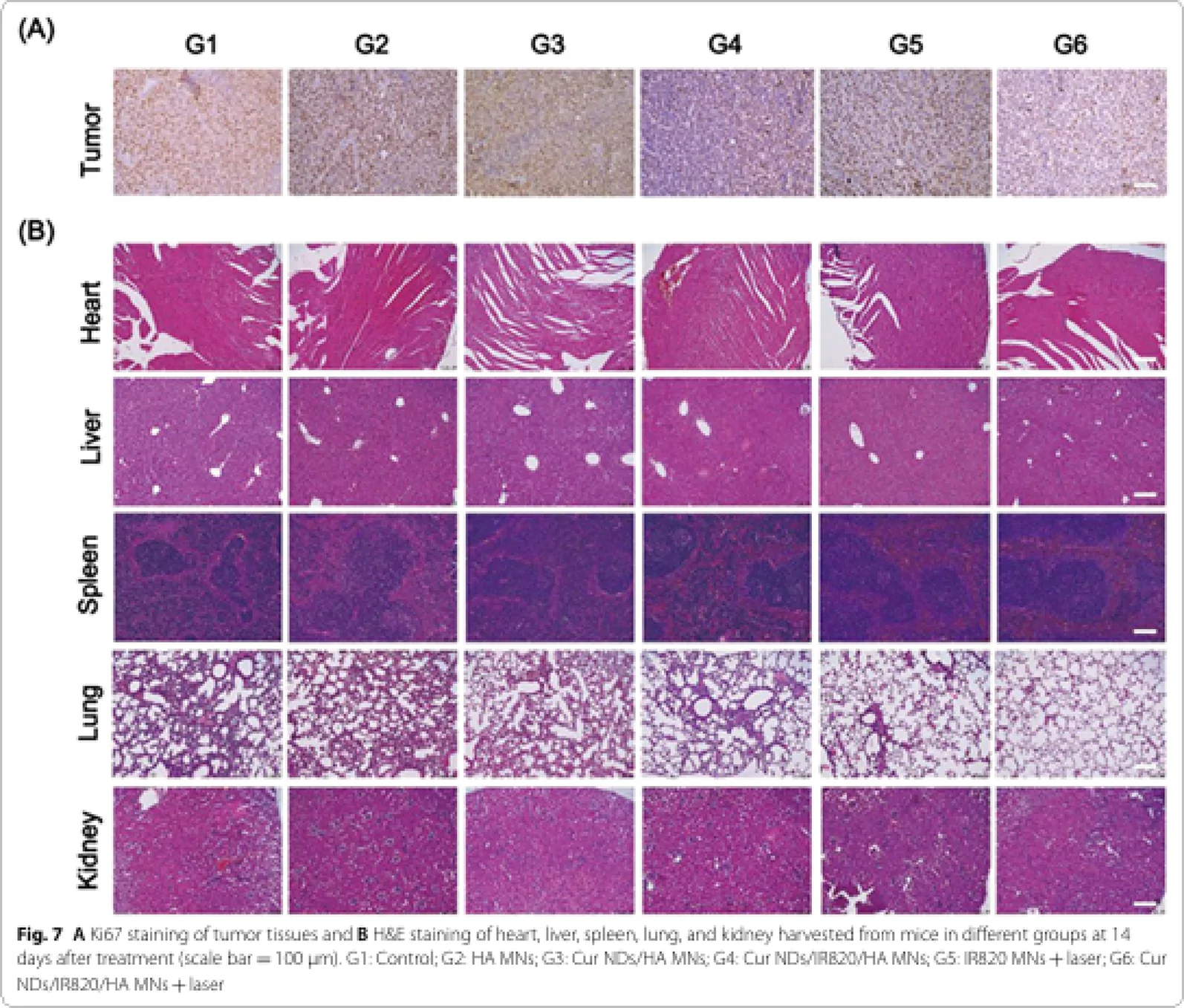

Correct Fig. 7.

**Fig. 7:**
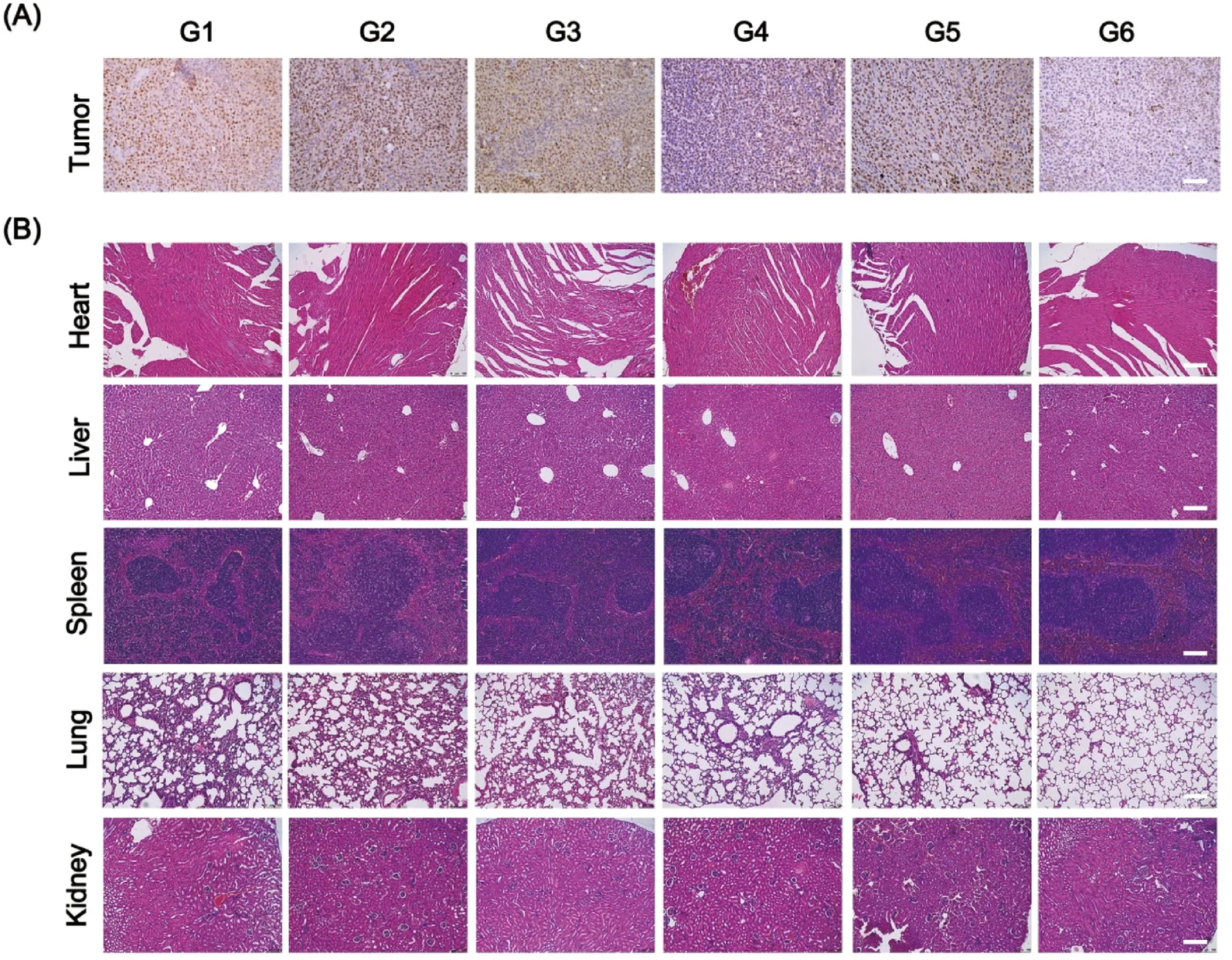
(**A**) Ki67 staining of tumor tissues and (**B**) H&E staining of heart, liver, spleen, lung and kidney harvested from mice in different groups at 14 days after treatment (scale bar = 100 μm). G1: Control; G2: HA MNs; G3: Cur NDs/HA MNs; G4: Cur NDs/IR820/HA MNs; G5: IR820 MNs + laser; G6: Cur NDs/IR820/HA MNs + laser

Incorrect Fig. 8.

**Figure:**
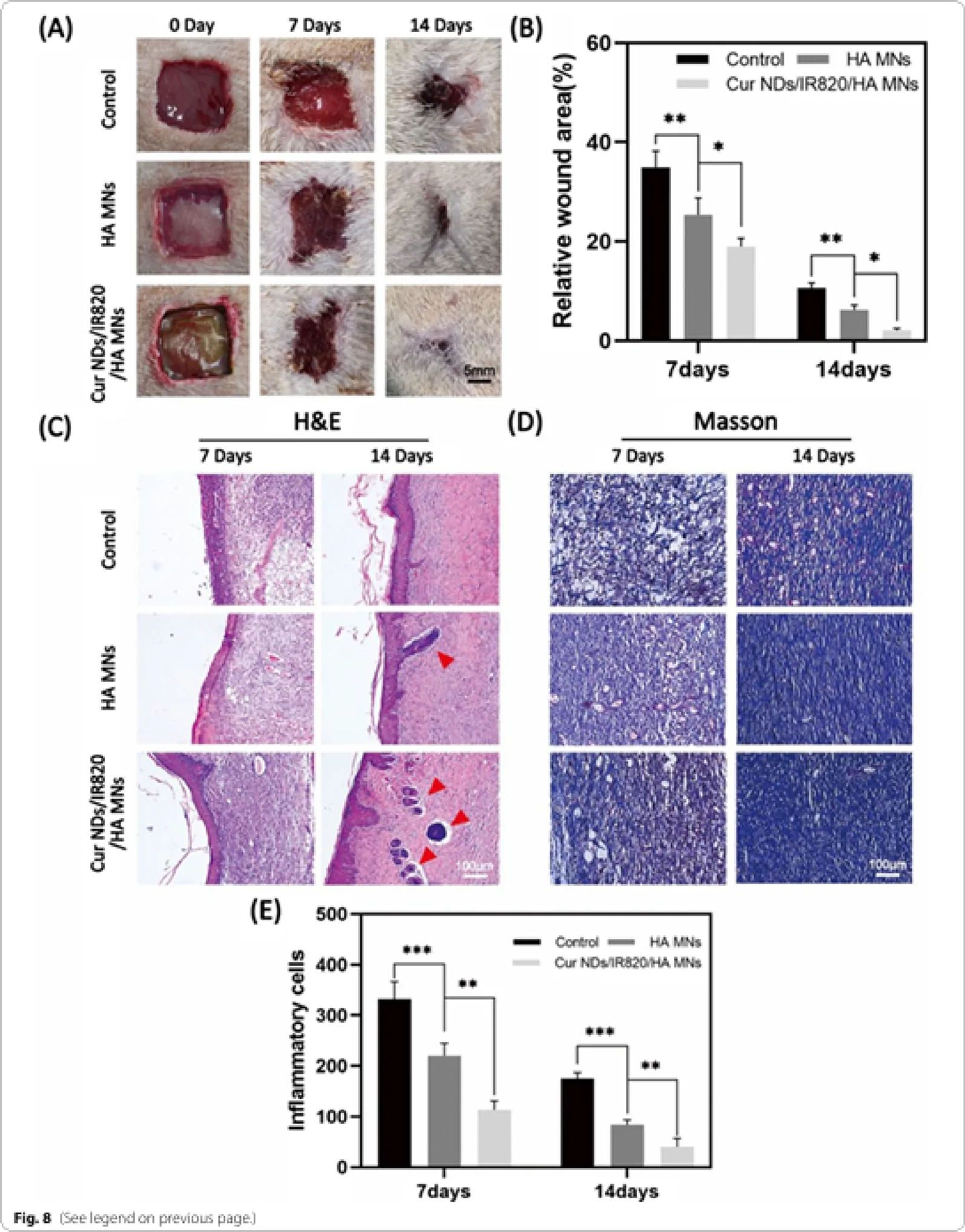

Correct Fig. 8.

**Fig. 8:**
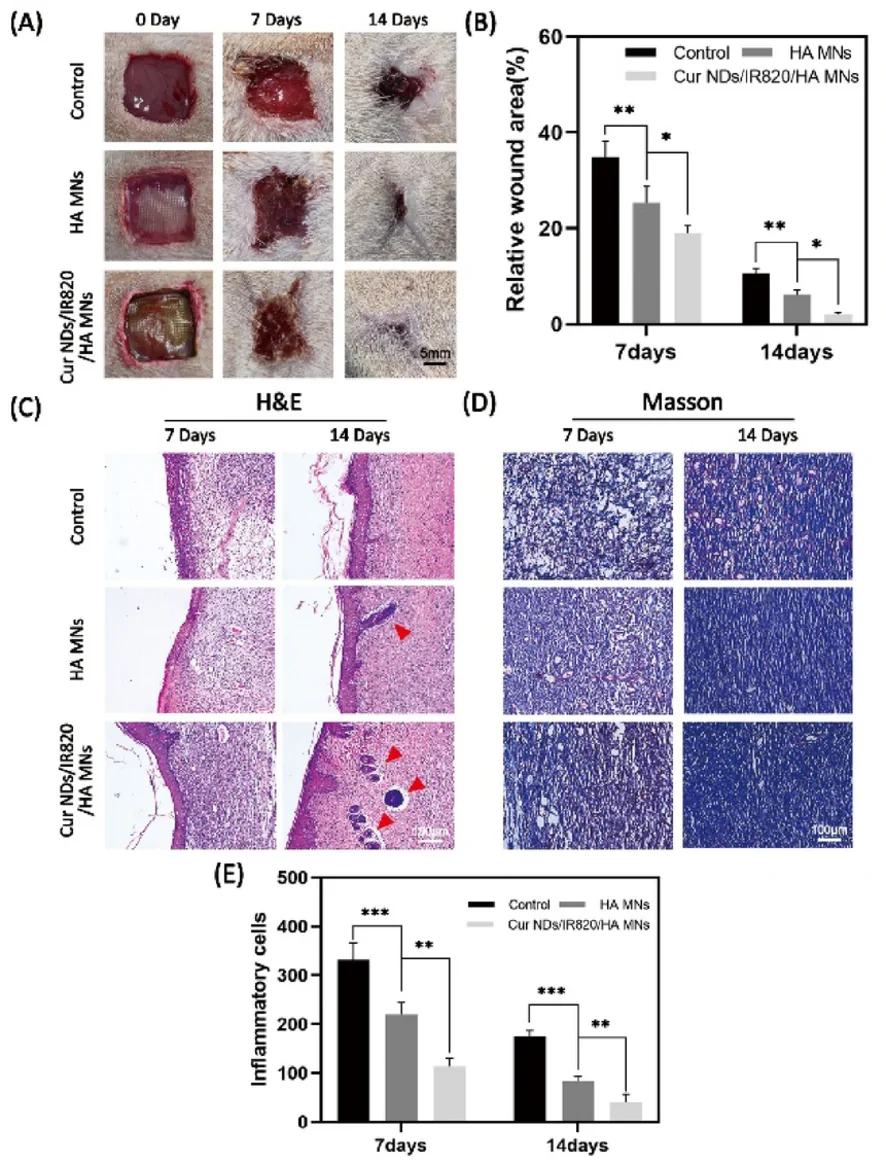
**A** Photographs of skin repair at 0 day, 7 days, and 14 days in control, HA MNs, and Cur NDs/IR820/HA MNs groups. **B** Relative wound area of control, HA MNs, and Cur NDs/IR820/HA MNs groups at 7 days and 14 days. **C** H&E staining images and **D** Masson staining images in control, HA MNs, and Cur NDs/IR820/HA MNs groups at 7 days and 14 days. The red arrows indicated newly formed skin appendages. **E** The statistical data of inflammatory cells at 7 days and 14 days. *p < 0.05, **p < 0.01, ***p < 0.001

The original article has been corrected.

